# The burden of waiting to access pain clinic services: perceptions and experiences of patients with rheumatic conditions

**DOI:** 10.1186/s12913-021-06114-y

**Published:** 2021-02-18

**Authors:** Simon Deslauriers, Jean-Sébastien Roy, Sasha Bernatsky, Nathan Blanchard, Debbie E. Feldman, Anne Marie Pinard, Mary-Ann Fitzcharles, François Desmeules, Kadija Perreault

**Affiliations:** 1 Center for Interdisciplinary Research in Rehabilitation and Social Integration (CIRRIS), Centre Intégré Universitaire De Santé Et De Services Sociaux De La Capitale-Nationale, 525, boulevard W.-Hamel, Quebec (QC), G1M 2S8 Canada; 2 Faculty of Medicine, Université Laval, Québec, Canada; 3 McGill University Health Centre (MUHC), Montreal, Canada; 4 McGill University, Montreal, Canada; 5 Research Institute of the McGill University Health Centre (RI-MUHC), Montreal, Canada; 6 Faculty of medicine, Université de Montréal, Montreal, Canada; 7 Centre for Interdisciplinary Research in Rehabilitation of Greater Montreal (CRIR), CRIR, Montréal, Canada; 8 Public Health Research Institute of Université de Montréal, Montréal, Canada; 9 Centre hospitalier universitaire (CHU) de Québec, CHUL, Quebec, Canada; 10 Maisonneuve-Rosemont Hospital (CRHMR) Research Center, CRHMR, Montreal, Canada

**Keywords:** Access, Waiting time, Pain clinics, Rheumatic conditions, Chronic pain

## Abstract

**Background:**

Extensive waiting times before receiving services is a major barrier to adequate pain management. Waiting times may have a detrimental impact on patients’ conditions and quality of life. However, there remains a lack of knowledge on the actual experiences of patients waiting to receive services, especially for those with rheumatic conditions. The present study aimed to gain an in-depth understanding of perceptions and experiences of patients with rheumatic conditions regarding access to pain clinic services. The secondary objective was to identify possible solutions to improve this access according to patients’ perspectives.

**Methods:**

This qualitative study based on semi-structured interviews was conducted with adults with rheumatic conditions waiting to access pain clinics in the province of Quebec, Canada. Interviews were transcribed verbatim and analyzed using thematic content analysis.

**Results:**

Twenty-six participants were interviewed (22 women and 4 men; mean age 54 ± 10 years). Four main themes were identified: 1) the perception that waiting time is unacceptably long; 2) how the lack of information affects patients’ experiences of waiting; 3) patients’ various expectations towards the pain clinic, from high hopes to disillusionment and 4) carrying an emotional, physical and financial burden resulting from the wait. Participants reported several solutions to improve the experience of waiting, including providing information to patients, increasing resources, improving prioritization processes and care coordination, and providing alternative interventions to patients during the wait.

**Conclusions:**

For patients with rheumatic conditions, access to pain clinic services is challenging due to extensive waiting times. The burden it imposes on them adds to the existing challenge of living with a chronic rheumatic condition. The solutions identified by participants could serve as building blocks to develop and implement measures to improve patients’ experience of accessing pain-related services.

**Supplementary Information:**

The online version contains supplementary material available at 10.1186/s12913-021-06114-y.

## Background

Chronic pain is an important cause of disability, affecting one in five individuals among North American and European populations [1–3]. For approximately 40% of these persons, pain is caused by a rheumatic condition [3]. Rheumatic conditions include a variety of diseases and syndromes related to autoimmune (e.g., systemic lupus erythematosus), inflammatory (e.g., rheumatoid arthritis) or degenerative conditions (e.g., osteoarthritis) as well as widespread body pain (e.g., fibromyalgia) [4, 5]. In persons with rheumatic conditions, having chronic pain is associated with a poorer health status [6–10], anxiety and depression [11, 12]. Because of the multidimensional nature of pain, its management often requires a multidisciplinary pain management approach [13, 14]. By combining pharmacological and non-pharmacological interventions (e.g., exercise, cognitive behavioral therapy, etc.), multidisciplinary pain management programs have been shown to lead to reduced pain intensity, better self-efficacy and higher quality of life in persons with rheumatic conditions [15, 16]. These programs have also been associated with a reduction of opioid use in patients with fibromyalgia [17].

However, access to multidisciplinary pain management programs is challenging and long waiting times have been reported in pain clinics in many countries [18–22]. A recent Canadian study conducted by our research team found that a third of patients living with a rheumatic condition waited more than 6 months to receive services in pain clinics, the average waiting time being 7.9 months (standard deviation = 10.2; median = 4.1) [23].

Delays in accessing services in pain clinics may have detrimental consequences on patients’ health and well-being. Waiting times longer than 6 months before receiving chronic pain treatment have been associated with worse health-related quality of life among patients with different chronic pain conditions [24] and a small but significant deterioration in psychological health [24, 25]. In addition to negative impacts during the waiting period, long delays also affect treatment outcomes once services are obtained. Patients with rheumatic conditions waiting less than 2 months before receiving treatment were found to have larger improvements in pain interference, pain intensity and health-related quality of life compared to patients who waited longer before their initial visit to the pain clinic [26]. While previous studies indicate a negative impact of waiting time on patients’ conditions, there remains a lack of knowledge on *how* and *why* patients are affected by the waiting period. Such questions are best answered by understanding patients’ perceptions and experiences shared through their own voices. Such knowledge is important to guide the development of effective and meaningful measures to improve patients’ experiences of access to services.

Thus, the aim of this study was to gain an in-depth understanding of perceptions and experiences of patients with rheumatic conditions regarding access to pain clinic services in the province of Quebec, Canada. The secondary objective was to identify possible solutions to improve patients’ experience of access to these services according to their own perspective.

## Methods

This study used a descriptive qualitative design [27, 28] and was part of a mixed-methods project aiming to document access to pain clinics for patients with rheumatic conditions [23, 26]. Data were obtained through semi-structured interviews. The study was approved by the *Institut de réadaptation en déficience physique de Québec* Ethics Committee (#EMP-2015-449). The study is reported in line with the COREQ guidelines (see Additional file 1).

### Study population

Study participants had to meet the following inclusion criteria: 1) aged 18 years or over; 2) diagnosed with a rheumatic condition (e.g., osteoarthritis, inflammatory arthritis, fibromyalgia); 3) able to communicate orally in French or English, and 4) waiting for an appointment in a pain clinic OR referred to a pain clinic OR received services in a pain clinic in the past 6 months in Quebec, Canada. This 6-month time frame was chosen to minimize recall bias [29]. A “pain clinic” was defined as a secondary or tertiary care setting specialized in pain management and was not limited to university affiliated or urban settings.

### Sampling and recruitment of participants

Participants were selected based on a combination of sampling methods that aimed to reach a diversified and comprehensive sample of patients with a broad set of perceptions and experiences regarding access to pain clinic services [30–32]. First, a convenience sampling approach was used by sending an email invitation through patients and academic-based organizations’ contact lists (i.e., university, Arthritis Society, Quebec Association of Chronic Pain) and posting an advertisement in pain clinic waiting rooms. Second, the snowball sampling method was used by inviting participants to suggest any other potential participants (e.g. relatives, colleagues). Lastly, a purposive sampling approach was used by asking pain clinic nurses and physicians to share the invitation to participate in the study to patients that fit a specific profile based on certain sociodemographic (age, gender), contextual (wait time duration) and clinical criteria (diagnosis). This method allowed to target participants with profiles underrepresented in the sample until then (e.g., men patients or patients with osteoarthritis).

Potential participants interested in the study contacted the research team by email or telephone; then, the objectives of the study were explained, the inclusion criteria were verified, and the interview scheduled. With an initial targeted sample of approximately 25 to 30 participants, recruitment continued until data saturation was reached, whereby no new theme stemmed from the interviews and the density and depth of the data were considered sufficient to answer the research objective [30, 31, 33].

### Data collection

Semi-structured interviews lasted on average 45 min (ranging from 15 min to 2 h) and were conducted by SD (MSc, physiotherapist, male, with qualitative research training) and NB (undergraduate student, male). Interviews took place either in person (at the participant’s home or the research centre) or over the phone, depending on participants’ place of residence and preference. An interview guide (see Additional file 2) was developed based on evidence from the literature and the quantitative results of the aforementioned mixed-methods project [23, 26]. The interview guide was pre-tested with two participants with rheumatic conditions and was adapted throughout data collection in an iterative process [34]. The questions pertained to perceptions and experiences of access to pain clinic services, the utilization of services while waiting, the impact of waiting time on participants’ conditions and potential solutions for improvement. Interviews were audio-recorded and transcribed verbatim.

### Data analysis

Data were analyzed iteratively during the data collection process to inform the recruitment and interview questions in order to gain an in-depth understanding of emerging themes [34]. Thematic content analysis was conducted using both deductive and inductive approaches [35, 36]. The deductive approach was based on broad categories pertaining to the study’s objectives, while the inductive approach allowed to identify new themes and subthemes derived from the data. According to Miles et al. steps of coding, the first cycle coding, or the data condensation phase, aimed to code words, sentences and paragraphs from the interviews and sort them into categories in a structured but flexible arrangement. The coding process included descriptive, emotion, values and evaluation coding techniques [36]. The code list evolved into a thematic arborescence as the data analysis progressed: codes were added, moved to other categories, combined or subdivided [36]. The first five and last two interviews were coded independently by two members of the research team (SD and NB) in order to discuss disagreements and reach a common understanding of the main codes and thematic arborescence, thus ensuring the validity and fidelity of the analysis. After approximately 15–18 interviews, most codes and categories were already identified and the remaining interview data allowed to reach a deeper understanding of the existing codes, explore variations between patients and expand the thematic arborescence into subcodes. Thus, data saturation was considered achieved [37]. The most relevant themes were retained and further analyzed in the second cycle coding, which aimed to identify relationships and explanations and derive an integrated synthesis of the data in order to gain a higher level of analysis [36]. The coding processes and the thematic analysis was conducted using NVivo 12 (QSR International Pty Ltd.) [36] Key citations were translated from French to English and presented to illustrate the main themes.

## Results

Twenty-six participants were interviewed (10 in person, 16 by telephone), 22 women and four men. The mean age was 54 years (standard deviation = 10 years), ranging from 39 to 82 years (see Table 1). Participants had a variety of education and annual household income levels, but only three (11.5%) were working (full-time or part-time). The most common rheumatic condition reported was fibromyalgia (69.2%), followed by osteoarthritis (42.3%); ten participants reported more than one rheumatic condition, including eight with a combination of osteoarthritis and fibromyalgia. The majority of participants (61.5%) were on a pain clinic waiting list, six (23.1%) had already received services, two (7.7%) were denied access after being referred to a pain clinic and two other participants (7.7%) had consulted before and were back on a waiting list after being referred again. Waiting time was 6 months or more for 20 (77%) of participants and 2 years or more for nine participants (35%).

**Table 1:** Participants’ clinical and sociodemographic characteristics

Gender	n (%)
Women	22 (84.6)
Men	4 (15.4)
**Age**	
< 40	3 (11.5)
40–55	12 (46.2)
56–70	10 (38.5)
> 70	1 (3.8)
**Education (highest completed level)**	
Elementary	1 (3.8)
High school	8 (30.8)
College	5 (19.2)
University	12 (46.2)
**Employment status**	
Employed (full-time or part-time)	3 (11.5)
Sick leave / on temporary disability	6 (23.1)
Sick leave / on permanent disability	5 (19.2)
Retired	10 (38.5)
Other	2 (7.7)
**Household income (CAD)**	
< 20,001	5 (19.2)
20,001-30,000	5 (19.2)
30,001-40,000	3 (11.5)
40,001-50,000	2 (7.7)
50,001-70,000	3 (11.5)
> 70,000	7 (26.9)
Missing	1 (3.8)
**Diagnosis**^a^	
Fibromyalgia	18 (69.2)
Osteoarthritis	11 (42.3)
Rheumatoid arthritis	3 (11.5)
Ankylosing spondylitis	6 (23.1)
**Pain duration** (years, mean ± SD)	9.2 ± 8.3
**Wait list status**	
On the wait list	16 (61.5)
Has consulted	6 (23.1)
Excluded from the list	2 (7.7)
Consulted before and back on wait list	2 (7.7)
**Wait time duration**^b^	
< 2 months	1 (3.8)
2–6 months	4 (15.4)
6–12 months	2 (7.7)
1–2 years	9 (34.6)
2–5 years	8 (30.8)
> 5 years	1 (3.8)

*SD* standard deviation

^a^More than one answer was possible

^b^Including patients on a wait list, those who consulted and those who were excluded

**Fig. 1:**
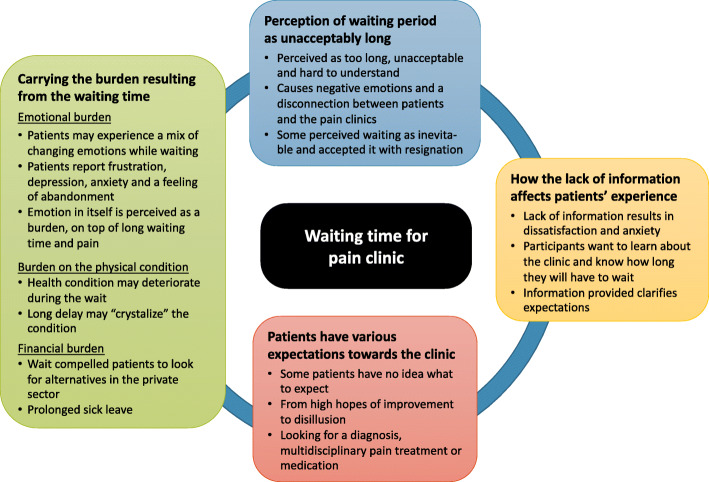
Participants’ experience of waiting to access to pain clinic

Four main themes emerged through the perceptions and experiences of access to pain clinic services expressed by the participants: 1) perceiving the waiting period as unacceptably long; 2) how the lack of information affects their experiences; 3) having various expectations towards the pain clinic, from high hopes to disillusionment and 4) carrying an emotional, physical and financial burden resulting from the wait. With the long waiting times at its core, the experiences of access were shaped by these four themes which were inter-related, as illustrated in Fig. 1.

### Participants perceived the waiting time as unacceptably long

The majority of participants shared negative perceptions of the waiting period. Many of them thought that waiting for many months and years was too long and was a difficult overall experience for them: “access to pain clinics is really too long” (A019), “too long because we are suffering right now, and the sooner we have access, the sooner we will at least have a few solutions [to treat pain]” (A023). Participants found it “very difficult to be in a dynamic of wait” (A001). The language used by some participants illustrates the emotions behind the negative perception of wait: “it is f***ing long” (A014), said a woman who had to wait for 2 years before her first appointment. Thus, participants viewed the waiting period as unacceptable: “These are people that suffer like martyrs and they leave them at home without support” (A014). They used various words to describe this time: “indecent” (A001), “abominable” (A012), “terrible” (A022) and “ridiculous” (A015). “It does not make sense,” said a woman who had been waiting 8 months (A011), a feeling of disbelief and dismay that was echoed by a few other participants: “I cannot believe that it is three years of waiting for the pain clinic, I cannot believe it” (A016). Another participant said: “It does not make sense to mess around with people like that.” (A014).

Additionally, some participants had been waiting for so long they did not believe they would receive services anymore. This led some participants to question whether the expected benefits of treatment at the pain clinic were worth the wait, and even disregarded the pain clinic as a possible option to manage their pain. As they were not able to count on the pain clinic to receive services due to extensive waiting time, participants reported a feeling of hopelessness: “I don’t have hope for the pain clinic. This is my feeling. I don’t hope, I don’t count on it. I don’t count on it, I told myself, ‘If I wait for this, I will never go back to work, I will never go back to my life’.” (A022).

The perception of the waiting time as an endless delay before treatment seemed to lower the expectations of obtaining positive outcomes from services offered at the pain clinic. As a result, some participants turned their back to the clinic and looked for alternatives.

Participants also reported a sense of distance between them and the pain clinic, due to long waiting times and lack of information: “I have the impression that I am in Europe and [the pain clinic], they are in Canada or I don’t know. And when I talk to others, all the people whom I met in chronic pain, [ …] it’s always that, [they feel] it’s far away.” (A004) This geographical distance metaphor illustrates the disconnection between the pain clinics and the patients’ needs.

Conversely, some participants seemed to accept the wait and perceived it as inevitable. This perception could be explained by previous experiences of waiting for other services that made it seem normal to wait for health care. The few participants who accepted the waiting period pointed to the large number of persons with chronic pain and the lack of human resources in the healthcare system. One participant noted: “It is maybe more the lack of personnel, we know it’s a major problem in Quebec, we know we have problems with personnel.” (A016) In some cases, however, the acceptance of waiting was preceded by a phase of negative feelings and psychological distress, as expressed by one participant: “I wait and I tell myself well, then we’ll just see what happens. When it will happen, it will happen. [ …] So you know, I don’t put emphasis on all of this anymore, I don’t spend negative energy anymore by making myself sick or crying or arguing on the phone or …” (A011). This participant also expressed a negative perception of the waiting period, suggesting a state of resignation to the situation rather than pure acceptance.

Only two participants described a positive perception of their waiting period, one who was satisfied to have his first appointment within 3 weeks and another woman who felt the delay allowed her to progress in the meantime and be more prepared when she would eventually access the pain clinic.

### How the lack of information affects patients’ experiences of access

According to most participants, the information provided by the pain clinic during the wait was often insufficient, and this issue was considered a key element of their experiences of access for pain clinic services. They reported insufficient information regarding the services they would receive from the pain clinic, the prioritization process used and the estimated duration of time they would have to wait. This lack of information affected patients’ expectations towards the pain clinic and contributed to the emotional burden related to waiting.

Most participants reported having received very little information regarding the pain clinic and wondered what type of services they would receive, how many appointments they would require, which healthcare providers they would meet or what benefit they should expect. This lack of information led to dissatisfaction among participants. One of them said: “you are like a number and they don’t keep you informed of the details, they don’t give you the information” (A012). This citation is an example of how participants reported being left alone and feeling unimportant to the healthcare system. Another participant expressed this feeling of abandonment due to the lack of information by saying: “there is no way to know anything [ …] it’s as if I did not exist at all” (A023).

Many participants also reported a lack of information regarding the wait list. Some did not receive any confirmation that their referral had been received or that their name was still on the pain clinic waiting list after a few months or years of waiting, which created an uncertainty that was a source of anxiety for many participants during the waiting period. They also wondered how patients were prioritized to access pain clinic services. While most participants understood that patients with more urgent needs should be treated first, they asked for transparency with the prioritization process in order to help them better accept the situation. For example, informing them on their priority level and the prioritization criteria could help them understand why they had to wait for so long. In addition, they did not know how long they would have to wait or what rank they were on the list, which prevented them from planning their waiting period based on the expected delay. For example, one participant would have looked for alternatives instead of waiting passively, had she known the delay would exceed 6 months. Information on the duration of wait was also perceived as valuable because it gave participants a sense of hope: “I wished I had known if my case was progressing, if my waiting period was reduced, if I was at 6 months, one month, someone to tell me I had 6 months left, something like that to encourage me.” (A003) On the other hand, participants who were told that they had to wait a few years felt demoralized and had lost hope of being admitted to the pain clinic. Similarly, being told that her case was considered as a low priority led to “extreme frustration” for a participant (A007). Some participants had heard of long waiting times from other patients, as one participant expressed: “The only thing I hear about the pain clinic is, my god the delay is so long. It is not even if they offer this or this or that, it’s the long delay. ‘Don’t send your referral there, the delay is long’” . (A030).

In response to the lack of information provided, participants reported searching on internet or calling the clinic, but the information obtained was minimal. Some participants were more active than others in asking for information: “I called once a month for a few months but they always answered the same thing, so at one point you get tired of it, I told myself, ‘what’s the point in calling?’” (A016) In some cases, the staff refused to provide information over the phone, such as estimated waiting duration or rank on the wait list, a situation that resulted in patient’s frustration.

### Patients have various expectations towards services at the pain clinic, from high hopes to disillusionment

Another theme that emerged as reflecting participants’ perceptions and experiences of access to services at a pain clinic was their expectations towards these services, which were very different among participants. These expectations influenced patients’ experiences of waiting to access pain clinic services since they shaped emotions and perceptions of waiting, especially if the wait extended to several months or years.

To start, several participants had no idea what to expect from the pain clinics in terms of services, outcomes, or healthcare providers, a consequence of the lack of information previously discussed. For some participants, the pain clinic represented the highest degree of expertise in pain management, where experts would provide advanced pain interventions and finally improve their condition after multiple other failed interventions. One participant illustrated this by saying: “They will give me, maybe give me a new medication, so look, I want that. But I know this is the last step, and after this is God, it is the sky. This is the last step.” (A001).

Participants also had various beliefs regarding the interventions they expected to receive at the pain clinic and their outcomes. Many were looking for advanced prescriptions of third line medication or injection. Others wished to avoid medication and hoped for alternatives, for example, guidance for the use of cannabis or multidisciplinary management that included rehabilitation and psychological services, moral and social support and regular long-term follow-up of their condition. Regarding the outcomes following interventions, most participants anticipated an improvement in pain intensity, fatigue or disability. Others expected the pain clinic to be able to specify the diagnosis, even after multiple specialists’ opinions (e.g. rheumatologist, physiatrist). This was particularly the case for fibromyalgia patients, for whom understanding their condition was a challenge.

Overall, expectations ranged from high hopes that the clinic would improve their pain condition (e.g., “waiting for the magic day to happen” (A005)), while others had very little hope to improve and felt disillusioned regarding the benefits the pain clinic services would have. These varying expectations and hopes affected for some patients the degree of anticipation to receive services and thus the perception of waiting. For example, a participant noted: “I had so little hope about what they would achieve to do or find, that I did not have, I was not waiting with anticipation that they would call me.” (A015) They also affected how they experienced the services at the pain clinic once they obtained them. A woman with high hopes to improve her condition with the pain clinic services had to wait a couple of years before receiving services that ended up not meeting her expectations. In her own words, she “waited two years for nothing” (A025).

### Carrying an emotional, physical and financial burden resulting from the waiting time

According to participants, the experiences of access to pain clinic services resulted in a burden in terms of emotions, consequences on the physical conditions and costs. This burden was mainly caused by the long duration of the waiting period but was also influenced by the lack of information from the pain clinics as well as participants’ expectations and perceptions.

#### Emotional burden

Participants reported an emotional burden resulting from the waiting period characterized by negative emotions such as frustration, distress, anxiety and a feeling of abandonment. These emotions sometimes overlapped, and often changed over the course of the waiting period. One participant said: “The journey has been so long just to get [referred] to the pain clinic, and then it is impossible to get access to services once you get there. It’s as if they would tell us, ‘This is your chance, and once you get there the door is closed’. So yes, there has been some revolt, some anger, there has been everything. Until the point when I told myself, ‘Now, the anger is eating me inside, this is what’s going to get me into depression’. So that’s it, because I am helpless.” (A022).

Frustration was expressed by many participants: “You shout a slang word because you wonder why they haven’t called back yet, and why … You know, and you suffer so much and you are there, still waiting for them. Why are they letting me suffer? You develop a kind of hate, not a hate but … a frustration.” (A012) This frustration was often associated with the lack of information mentioned earlier. A man reported being frustrated, “because of the system, because of the way people are treated, because of the ignorance.” (A003).

Some participants mainly shared feelings of distress and sadness relative to the waiting period. One woman expressed how waiting time affected her: “this waiting disrupts your whole life, it is depressing” (A004). Another participant qualified a 3-year waiting period as demoralizing and chose to take this period 1 day at a time, just “like people who are quitting drinking” (A005). Some participants were emotional during the interview, and a few cried or held back tears.

For others, the waiting time was a source of stress and anxiety. For example, one woman wondered every time she received a phone call if it was the pain clinic calling. She was also worried to miss a phone call whenever she was away from home. For one participant, the anxiety was also related to uncertainty in the duration of the wait and in the outcome of the eventual treatment. A participant noted that patients with chronic pain “don’t really need stress, because it increases the pain, it increases the fatigue.” (A023).

The feeling of “total abandonment” (A003 and A019) was strongly related to the lack of information, the absence of follow-ups during the wait and the patients’ lack of control on their wait list situations. Feeling abandoned translated into a feeling of being “completely left alone” (A003) and being meaningless. As one participant said: “You know, you look at a chessboard, that’s it. We are the little pawn; we are not the knight or the queen or the king or anything. We are the little pawn and we die quickly in that because the little pawn, at one point, we remove them, we put them aside.” (A004) This feeling of abandonment was especially salient for a participant who reported being removed from the wait list after having waited several years. She felt like “something, I don’t know, that we put aside, a rag” (A004).

#### Burden on the physical condition

Some participants, essentially those who were waiting more than 6 months, reported a deterioration of their health condition during the wait, such as a worsening of the pain intensity, quality of life, fatigue or activity limitations. A participant noted: “There are always consequences, because everything deteriorates [ …] one, it’s the pain, two, the chronic fatigue related to the pain, three, my capacity to concentrate, to have fun, just some fun in my life.” (A028). One woman said her physical capacity decreased to the point she needed a walking aid to go out. Another participant linked the increasing frequency of pain flare ups of her rheumatic condition with stress factors such as the waiting period. Conversely, one participant noted: “In my case, I am not more affected you know, in the sense that the disease did not progress faster. It progressed normally at its rhythm, but I am not worse physically” (A011). Yet, a few participants noted the importance of early pain management to slow down or stop the degenerative decline of chronic conditions such as rheumatic diseases. They wondered, “What if my pain had been managed from the beginning?” (A016), “Would I be at the point where I am right now? Would my pain have developed into fibromyalgia?” (A004). It was also mentioned that longer pain duration leads to higher risk that the condition “crystalizes”: “The more it goes, the worse it gets. And I won’t regain the flexibility when the … if I can finally have a treatment that works to stop or slow down the disease. But the damage that I have, it will stay there.” (A015).

#### Financial burden

The extensive waiting time compelled patients to look for alternatives to pain clinic services to treat their pain, including in the private sector such as physiotherapy, acupuncture, psychology or physiatry. Paying for services, however, added a significant financial burden to some participants, and this was not an option for every one of them: “I would have gone to private services, but now I mean, I am cut to the throat. I am even thinking of selling the house, I don’t have a choice.” (A028) One participant considered borrowing money to pay for private services, while another said, “I have to look elsewhere and pay from my pocket for other treatments as well. So I impoverish at the same time. Not only have I been on sick leave for 20 months, but financially I am sinking.” (A022).

Also, the waiting time was perceived as extending the duration of sick leave or work disability status in some cases, which can have important financial impacts. One participant explained: “The consequences, well it’s because if the pain management [clinic] could give me a response, and that it would go faster, I would not still be on sick leave, probably.” (A013) Another participant noted: “It sure has an enormous impact on people not to have access to resources quickly [ …] they are not able to work anymore, they have to go bankrupt, to lose their house …” (A015).

### Solutions identified by participants

Participants identified various solutions to improve the experience of access which included providing more information to patients, improving the prioritization of referrals and the coordination of care, increasing resources in pain clinics and offering alternative intervention during the wait.

#### Providing information

One key solution was directly related to one of the themes previously discussed, that is the lack of information. Indeed, many participants suggested that pain clinics should provide more information to patients in order to clarify expectations towards the clinic and facilitate the waiting period. Such information should also be shared to family physicians, who could inform their patients at the time of referral. Participants suggested explaining what a pain clinic is, its goal and the expected outcomes. This could help patients avoid unrealistic expectations (for those with unrealistic high hopes) or understand why it is worth the wait (for those who stopped believing). A man participant said that patients who know what to expect would be more likely to look forward to receiving services at the pain clinic. Information provided to patients could also help reduce the stress and anxiety associated with the uncertainty of the waiting period. A simple phone call from the pain clinic could have an impact: “Anyway, me and the others too would find it maybe a little less long, the feeling abandonment, if we could be called.” (A008). Participants suggested being transparent with the expected duration of the waiting time. A phone call or an email telling them that they are moving up on the wait list and that their waiting time is decreasing could reassure and “comfort” patients, even if they are being told they still have 6 months left to wait. It could also “encourage” patients during the wait: “at least you see a delay, there is something at the end” (A017). One participant made a comparison with people waiting in line and noticing their turn is about to come: “it has a psychological effect, it fills you up with good mood, you tell yourself, ‘well finally it’s my turn’.” (A016) An estimation of the duration of the wait may also encourage patients to search for alternative treatments during the wait: “Me, personally it would tell me: ‘Ok, I have to wait for two years. Yes, it’s a little bit demoralizing, but however, in the meantime, what do I do?’ So the decision comes down to me, to do something.” (A017).

In addition, a participant suggested to explain the reasons of the long waiting time. For example, knowing the way referrals are prioritized and the number of staff versus the number of referrals may help patients understand and accept their situation of waiting, or at least resign to it. Many participants would have appreciated a simple confirmation that their referral had been received by the pain clinic and a periodic follow-up that they were still on the wait list. One participant perceived that such practice was part of elementary customer service standards that are common in other sectors outside of health care and should be expected in pain clinics. A participant also suggested that a periodic follow-up could allow to update the patient’s condition and maybe change the priority accordingly. In terms of format, participants suggested information could be communicated to patients by different means: phone calls, information booklets, emails, postal mail, websites, group sessions during the wait, etc.

The information participants had wished to know included:

- details about the referral (diagnosis written on the referral, date the referral was sent and to which clinic)
- a confirmation that the pain clinic had received their referral
- their rank on the list or their category of priority
- the prioritization process and criteria to determine the priority
- estimation of the duration of wait
- periodic follow-up to confirm their name is still on the list
- services provided by the pain clinics, healthcare providers available, interventions, the facilities
- expected clinical outcomes of the pain clinic and the goals of the intervention
- what to expect during the first appointment the number and frequency of appointments

#### Prioritization of referrals: give the patients a voice

Participants recommended improving the prioritization process by allowing patients to explain their condition and specific needs to complement the physician referral. A participant regretted that “someone decided that our priority is low and we don’t have any news for years [ …] there must be at least an evaluation with the patient …” (A003). Two participants compared it to the triage at the emergency department, where patients are evaluated before being given a priority level. Participants suggested various methods to take into account patients’ perspectives, either with an in-person evaluation, a telephone interview or by filling out a form.

#### Improve coordination and organization of care

Better coordination of care was also suggested as a way to improve patients’ experiences of access. For example, improving communication between physicians would allow them “to collaborate and push towards the same objective, that is helping the patient reach the pain clinic” (A022). A better communication between the referring physician and the pain clinic could possibly result in an earlier referral, better prioritization, or redirection towards alternatives to the pain clinic if necessary. A participant said: “because if they talk to each other more, maybe we will have a place [at the pain clinic] more quickly, maybe there will be more clinics and maybe we will have more solutions.” (A004) Some participants also needed to be oriented around the healthcare system. One participant wished someone would have told her “‘Ok, now, you need to do this, you need to go there’ Because me, I think I tried by myself many things and now, I don’t know what to do anymore” (A005).

A woman recommended having different levels of treatment at the pain clinic, for example a first line of physical therapy while patients wait for the second stage of the pain clinic trajectory, similar to a primary, secondary and tertiary care structure. With a preliminary treatment in place during the wait, “there would already be a faster management, the patient maintains hope” (A022). Similarly, two participants mentioned the need to establish smaller satellite clinics that would serve as a first line of services for patients with chronic pain. A woman noted that primary care services are not adapted to treat chronic pain and specialized services are not accessible, and that there is no in-between. Satellite clinics distributed across the province could also make pain treatment more geographically accessible. In addition, a centralized point of entry for referrals was suggested as a strategy to help orientate patients to the appropriate resource and to the clinic with the shortest waiting time.

#### Increase pain clinics’ resources

A frequent and straightforward solution mentioned by participants to improve access was to increase the resources at the pain clinics by hiring more staff (e.g., physicians) in order to meet the demand. For some participants, it was obvious that the number of physicians had to be sufficient enough to manage the high number of referrals: “They must find new physicians, find money, work more hours per week, meet some people, ask the government for money, they have to work things out to deal with the demand.” (A003). On the other hand, some participants acknowledged that there is a shortage of human resources in many areas of the healthcare system and that increasing resources would be easier said than done.

#### Alternative interventions during the wait

As previously mentioned, many patients had to find alternatives to treat their pain as a consequence of the long waiting time. In line with this issue, participants recommended that pain clinics provide or refer patients to alternative resources to help them cope with their pain during the waiting period. These alternatives included other healthcare services (rehabilitation, psychology), a booklet with physical exercises or advice to manage their pain (pain education, nutrition, mindfulness techniques). A woman suggested providing “psychological support from the beginning, it’s essential, otherwise the waiting, it’s a torture” (A022). A participant also suggested that these alternatives could be provided at the time of an initial evaluation appointment, similarly to pre-operative rehabilitation. Another alternative was group sessions during the wait to inform and educate patients with similar conditions.

Among the other resources that patients could be referred to during the wait, associations of patients with chronic pain or arthritis were frequently mentioned by the participants. These organizations were perceived to be an important complement to the healthcare system, as they could provide conferences, workshops and webinars to support people living with pain (e.g., nutrition advice, yoga training, etc.). One man said it is the association that “did the most to help me, it is not the hospital”. (A003) He was very grateful for the support received by the association while he was waiting to access the pain clinic. Patient organizations, through events and social media activity, also helped reduce isolation and allowed patients to share their experiences. They create a sense of community, which could be particularly useful for patients during the waiting period. Participants suggested that pain clinics could coordinate with these organizations or at least share their contact information to patients, especially since primary care practitioners were not aware of these resources, according to participants.

## Discussion

This study aimed to gain knowledge on perceptions and experiences of patients with rheumatic conditions regarding access to pain clinic services. It allowed to understand *how* and *why* patients are affected by the waiting period. In addition, participants shared potential solutions to improve patients’ experiences of accessing pain clinic services, a unique contribution of this study.

### Participants’ experiences

Overall, the results revealed that most participants reported a difficult experience of accessing pain clinics. Long waiting times were at the core of this negative experience, which was characterized by four interrelated themes: 1) perceiving the waiting period as unacceptably long; 2) how the lack of information affects their experiences; 3) having various expectations towards the pain clinic, from high hopes to disillusionment and 4) carrying an emotional, physical and financial burden resulting from the wait. For instance, the lack of information received during the waiting period seemed to have influenced patients’ expectations towards the clinic and their emotions towards the waiting time, and in turn, the expectations affected the emotional burden resulting from the waiting period.

A main theme characterizing participants’ experiences of access to pain clinic services was their negative perception of waiting time, which they considered to be unacceptably long. Participants condemned this situation, where vulnerable patients are put on hold for a long period of time. Such delays to access pain clinics were also reported as a source of dissatisfaction in a UK study, in which waiting time was perceived as a main barrier to access [38]. The negative perception of waiting time could lead to lower expectation towards the clinic. This could explain why some felt that they could not count on the pain clinic to manage their pain and therefore tried to find alternative treatments.

The lack of information was an important issue reported by participants’, which notably resulted in anxiety and unrealistic expectations. Similarly to our findings, Gjesdal et al. also found that patients receiving chronic pain care criticized the lack of information on available treatment and noted that primary care providers themselves had little knowledge of the available treatment options for chronic pain [39]. Thus, many patients may have been referred to the pain clinic by their family physician without being told what the pain clinic actually was. This could explain why the lack of information was a major complaint by the participants. A qualitative study by Nøst et al. identified the lack of information as part of patients’ experience in Norwegian pain clinics and described findings similar to ours [40]. The authors found a relation between the insufficient information provided to patients and their diverse expectations towards the clinic [40]. Also, similar to our finding, some participants had no expectations that they would benefit from the pain clinic services, while others had greater expectation due to the high level of expertise of these services [40]. However, Nøst et al.’s study did not focus on the experience of *waiting* for services but rather focused on the experience of patients *attending* the pain clinic.

Thus, waiting time, lack of information and expectations towards the clinic were related to one another and characterized participants’ experience of access. For example, patients who anticipate a major decrease in their pain level upon receiving services from the pain clinic may be seriously disenchanted when they find out such improvement is typically minimal [41], especially if they contemplated this expectation for several months or years. On the other hand, a patient who maintained low expectations towards the pain clinic for a long period of time could potentially lack the necessary motivation to invest in pain treatments (e.g., physical exercise, learning self-management interventions). Thus, providing sufficient information to clarify patients’ expectations and reduce their anxiety and feeling of abandonment should be a priority for pain clinics.

In our study, participants carried an emotional, physical and financial burden resulting from the wait. This burden can be particularly challenging for patients with rheumatic conditions, who also have to bear the burden of their chronic pain, where pain is often accompanied by loss of mobility, fatigue, sleep disorders and psychological distress [8, 9]. With a mean pain duration of 9 years, such distress and suffering likely already had a significant impact on participants’ lives. Participants’ experiences of access to pain clinic services were characterized by multiple negative emotions, including frustration, sadness, anxiety and a feeling of abandonment. This finding is supported by the literature on the experience of waiting for surgery [42–44]. For instance, in a qualitative study of 32 patients awaiting orthopedic or cardiac surgery in Canada, Carr et al. found that patients frequently reported frustration regarding waiting time, especially from patients who expected great benefit from the surgery and those with worst symptoms [45]. Patients also experienced psychological distress resulting from the uncertainty regarding their place on the list [45].

Waiting time also imposed an additional burden on participants in terms of potential deterioration of their physical health, which in turn led to psychological distress, for example by exacerbating anxiety and frustration. This is in line with the findings from Lynch et al.’s systematic review on the consequences of waiting for chronic pain treatment, which describes a deterioration in psychological status and quality of life in patients waiting 6 months or longer [24]. The authors suggested that this amount of time could be a threshold beyond which waiting time becomes unacceptable [24]. In our study, participants who reported a deterioration of health had waited more than 6 months, which support Lynch et al.’s statement [24].

The burden resulting from the waiting time also included financial consequences, as some participants needed to look for alternatives to the pain clinic, often in the private sector, although not everyone was able to afford such services. This situation was also reported in other studies on pain clinic services or other publicly funded healthcare services (e.g., cardiac rehabilitation) in the UK, in which patients considered that opting for private sector was sometimes the only option when facing long delays [38, 46]. However, paying for private services had a serious financial impact on patients of lower socioeconomic status, who often are unable to work due to their pain condition. It is likely that opting for private services was a challenge for many participants, since approximately 40% of our sample had an annual household income less than 30,000 CAD, just above the viable income threshold [47].

While further research is needed for an in-depth ethical analysis of access to pain clinics, the findings of this study raise potential ethical issues. For example, patients rarely understand the prioritization processes and the reasons why they have to wait for so long. There is a need for more transparency in this regard, as recommended by Daniels & Sabin in their *Accountability for Reasonableness* (A4R) framework [48]. Also, patients are left on waiting lists for months and years without even understanding what to expect from pain clinics. Thus, pain clinics could share more information with patients in order to correct misconceptions, encourage realistic expectations and avoid disillusion or disappointment.

### Solutions

The present study highlights the need to reduce waiting time as well as to improve patients’ experience of waiting. Participants identified several solutions in this regard, including sharing more information with patients in order to reduce anxiety and clarify expectations towards the clinic and the services offered. For example, providing patients with an estimation of the duration of wait was found to reduce uncertainty and anxiety in patients waiting for surgery [45]. This solution was also in line with the results from McIntyre et al.’s review on policy implications and potential strategies to address the burden of waiting for health care [49], which concluded that greater transparency was key to resolve the barriers to access [49].

Participants also emphasized the need for a better coordination between pain clinic and primary care. This aspect was reported in another qualitative study by patients receiving chronic pain treatment as well as nurses from pain clinics in Norway [39]. In some of these clinics, family physicians were hired in order to improve coordination with primary care [39]. For example, educating primary care providers about the objectives and function of pain clinics could help reduce inappropriate referrals. Participants in that study also suggested designating a coordinator to facilitate the trajectory and communication between primary care and the pain clinic, in order to optimize continuity of care and patient follow-up [39]. This could be especially relevant to support patients during the waiting period, for example by empowering primary care providers to arrange alternative treatments in the meantime. In this regard, technologies such as eConsult, an online asynchronous consultation platform, could be particularly useful to facilitate communication between family physicians and specialists [50, 51]. Results from a study of patients from a pain clinic in the UK indicate that the lack of communication between physicians sometimes led to unnecessary referrals, contradictory diagnostic opinions as well as patients’ frustration and confusion [38]. Coordination of care is important for patients with chronic conditions, who often seek care from multiple healthcare providers [52]. Improving chronic pain services from primary to tertiary care and optimizing trajectories of care is especially important for patients with fibromyalgia. While some authors consider that most of these patients could be treated in primary care [53], the large proportion of patients with fibromyalgia in pain clinics may also illustrate the challenges in treating this condition in primary care, leading to frequent referrals for specialized services [23]. Moreover, evidence suggests multidisciplinary approaches are effective for the treatment of patients with fibromyalgia [15], yet, to our knowledge, such chronic pain approaches are not common in primary care. For these reasons, pain clinic services remain relevant for patients with fibromyalgia, at least until chronic pain treatments in primary care are improved. Another solution suggested by participants was to increase pain clinics’ capacity by hiring more staff. The lack of resources was stressed as a key issue by pain clinic nurses in Norway [39].

Finally, participants suggested providing alternatives for patients on the wait list, such as rehabilitation or psychological services, physical exercises, advice to manage their pain or information on patient associations. In a survey of a pain clinic in Ontario, almost half of patients waiting for services reported receiving written information on chronic pain management or being informed of local patient organization [54]. Such interventions could be provided through e-Health interventions specifically designed for patients with arthritis and chronic pain, especially considering the development of such technology during the COVID-19 pandemic. This could improve access to alternatives that may help alleviate the burden of waiting while keeping patients in an active state towards the management of their condition. Thus, they may be more prepared and motivated once they are admitted to the pain clinic.

### Limitations

A first limitation of this study is the small number of men included in the study, which limited gender-based analyses. However, such imbalance is consistent with the characteristics of pain clinics population, in which two thirds of patients with rheumatic conditions are women [23]. Second, there was a minority of participants who presented inflammatory arthritis (e.g., rheumatoid arthritis) and it is possible that we did not capture any specific perceptions and experiences of this sub-population of patients. Combined with the high proportion of participants with fibromyalgia, it is possible that this sample was not representative of the average rheumatology population. Third, the study included only a minority of participants with short waiting times and thus may not reflect their spectrum of patients’ experiences. This may represent a potential selection bias. Patients with shorter waiting times may not experience the same burden and negative perceptions towards access as those reported in the present study. Fourth, one interviewer (SD) had prior experience working as a physiotherapist with patients referred to pain clinics and having to wait several months or years to receive services, which could be associated with potential bias or assumptions. However, this experience also provided a better understanding of the research topic. Lastly, the present study was conducted in the context of a publicly funded healthcare system facing extensive waiting times and its findings may not transfer to other contexts.

## Conclusion

Long waiting times represent a barrier to access pain clinic services in Canada. In this study, most participants reported a difficult experience of accessing pain clinic services. Their experiences were characterized by a perception that waiting time is unacceptably long, a lack of information provided to patients, diverse and sometimes unrealistic expectations towards the pain clinics and an emotional, physical and financial burden endured by patients. For patients with rheumatic conditions, the difficulty in accessing pain clinics along with the burden it imposes on them added to their existing challenge of living with their chronic condition. Nonetheless, according to participants, pain clinics could improve patients’ experience by sharing more information with them, by improving prioritization processes, by ensuring a better coordination of care and by redirecting patients to alternative treatment options during the wait.

The findings of this study should be taken into account by managers and decision makers concerned with access to pain clinics [55]. The results should serve as building blocks to implement measures to shorten waiting times and reduce the burden associated with the wait, with the aim of improving patients’ experience of access to pain clinic services. On a larger scale, there is a need for more coordinated, comprehensive and systematic care trajectories for patients with rheumatic conditions and chronic pain, which include improved primary care services, earlier consultation to pain specialists (e.g., through eConsult) and better support for patients waiting for pain clinics. Patients’ voices provide valuable information and should be considered in the revision of the care pathway.

## Supplementary Information

**Additional file 1.** Table S1. Consolidated criteria for reporting qualitative studies (COREQ): 32-item checklist.

**Additional file 2. Interview guide**

## Data Availability

Access to de-identified interview verbatim transcripts may be considered upon request to the corresponding author.

## Abbreviation

CAD: Canadian dollar

## References

[1] Park J, Mendy A, Vieira ER (2018). Various types of arthritis in the United States: prevalence and age-related trends from 1999 to 2014. Am J Public Health.

[2] Reis C, Viana Queiroz M (2014). Prevalence of self-reported rheumatic diseases in a Portuguese population. Acta Reumatol Port.

[3] Schopflocher D, Taenzer P, Jovey R (2011). The prevalence of chronic pain in Canada. Pain Res Manag.

[4] van der Heijde D (2018). Common language description of the term rheumatic and musculoskeletal diseases (RMDs) for use in communication with the lay public, healthcare providers and other stakeholders endorsed by the European league against rheumatism (EULAR) and the American College of Rheumatology (ACR). Ann Rheum Dis.

[5] Barbour KE (2017). Vital signs: prevalence of doctor-diagnosed arthritis and arthritis-attributable activity limitation - United States, 2013-2015. MMWR Morb Mortal Wkly Rep.

[6] Sarzi-Puttini P (2015). The impact of pain on systemic rheumatic diseases. Best Pract Res Clin Rheumatol.

[7] Sarzi-Puttini P (2014). Pain in rheumatoid arthritis: a critical review. Reumatismo.

[8] Taylor P (2010). Patient perceptions concerning pain management in the treatment of rheumatoid arthritis. J Int Med Res.

[9] Neogi T (2013). The epidemiology and impact of pain in osteoarthritis. Osteoarthr Cartil.

[10] Perruccio AV, Power JD, Badley EM (2005). Arthritis onset and worsening self-rated health: a longitudinal evaluation of the role of pain and activity limitations. Arthritis Rheum.

[11] Odegard S (2007). Pain and psychological health status over a 10-year period in patients with recent onset rheumatoid arthritis. Ann Rheum Dis.

[12] Edwards RR (2011). Pain, catastrophizing, and depression in the rheumatic diseases. Nat Rev Rheumatol.

[13] Scascighini L (2008). Multidisciplinary treatment for chronic pain: a systematic review of interventions and outcomes. Rheumatology.

[14] Kudrina I, Shir Y, Fitzcharles MA (2015). Multidisciplinary treatment for rheumatic pain. Best Pract Res Clin Rheumatol.

[15] Hauser W (2009). Efficacy of multicomponent treatment in fibromyalgia syndrome: a meta-analysis of randomized controlled clinical trials. Arthritis Rheum.

[16] Finney A (2016). Multidisciplinary approaches to managing osteoarthritis in multiple joint sites: a systematic review. BMC Musculoskelet Disord.

[17] Hooten WM (2007). Treatment outcomes after multidisciplinary pain rehabilitation with analgesic medication withdrawal for patients with fibromyalgia. Pain Med.

[18] Peng P (2007). Challenges in accessing multidisciplinary pain treatment facilities in Canada. Can J Anaesth.

[19] Triva P, Jukic M, Puljak L (2013). Access to public healthcare services and waiting times for patients with chronic nonmalignant pain: feedback from a tertiary pain clinic. Acta Clin Croat.

[20] Hogg MN (2012). Waiting in pain: a systematic investigation into the provision of persistent pain services in Australia. Med J Aust.

[21] Fashler SR (2016). Systematic review of multidisciplinary chronic pain treatment facilities. Pain Res Manag.

[22] Siddiqui Q, Rangaswamy G (2013). *Waiting times for access to a UK multidisciplinary chronic pain service: how do we comply with IASP recommendations?* Pain news. Br Pain Soc.

[23] Deslauriers S (2019). Factors associated with waiting times for persons with rheumatic conditions in multidisciplinary pain treatment facilities. J Pain Res.

[24] Lynch ME (2008). A systematic review of the effect of waiting for treatment for chronic pain. Pain.

[25] Choiniere M (2010). The Canadian STOP-PAIN project - part 1: who are the patients on the waitlists of multidisciplinary pain treatment facilities?. Can J Anaesth.

[26] Deslauriers S (2020). The association between waiting time and multidisciplinary pain treatment outcomes in patients with rheumatic conditions. BMC Rheumatol..

[27] Sandelowski M (2000). Whatever happened to qualitative description?. Res Nurs Health.

[28] Sandelowski M (2010). What's in a name? Qualitative description revisited. Res Nurs Health.

[29] Coughlin SS (1990). Recall bias in epidemiologic studies. J Clin Epidemiol.

[30] Bourgeault IL, Dingwall R, De Vries RG (2013). The SAGE handbook of qualitative methods in health research.

[31] Sandelowski M (1995). Sample size in qualitative research. Res Nurs Health.

[32] Morse JM (2015). Critical analysis of strategies for determining rigor in qualitative inquiry. Qual Health Res.

[33] Nelson J (2016). Using conceptual depth criteria: addressing the challenge of reaching saturation in qualitative research. Qual Res.

[34] Pope C, Ziebland S, Mays N, Mays CPaN (2007). Chapter 7 - Analysing Qualitative Data, in Qualitative Research in Health Care.

[35] Hsieh HF, Shannon SE (2005). Three approaches to qualitative content analysis. Qual Health Res.

[36] Miles MB, Huberman AM, Saldana J (2014). Qualitative Data Analysis. A Methods Sourcebook.

[37] Francis JJ (2010). What is an adequate sample size? Operationalising data saturation for theory-based interview studies. Psychol Health.

[38] Hadi MA (2017). 'Treated as a number, not treated as a person': a qualitative exploration of the perceived barriers to effective pain management of patients with chronic pain. BMJ Open.

[39] Gjesdal K, Dysvik E, Furnes B (2019). Mind the gaps: a qualitative study combining patients’ and nurses’ reflections on pain care. SAGE Open Nursing.

[40] Nøst TH, Steinsbekk A (2019). ‘A lifebuoy’ and ‘a waste of time’: patients’ varying experiences of multidisciplinary pain centre treatment- a qualitative study. BMC Health Serv Res.

[41] Pagé MG (2017). Predicting treatment outcomes of pain patients attending tertiary multidisciplinary pain treatment centers: a pain trajectory approach. Canadian J Pain.

[42] Johnson EC, Horwood J, Gooberman-Hill R (2014). Conceptualising time before surgery: the experience of patients waiting for hip replacement. Soc Sci Med.

[43] Fitzsimons D (2003). Patient anxiety while on a waiting list for coronary artery bypass surgery: a qualitative and quantitative analysis. Heart Lung.

[44] McCormick KM, Naimark BJ, Tate RB (2006). Uncertainty, symptom distress, anxiety, and functional status in patients awaiting coronary artery bypass surgery. Heart Lung.

[45] Carr T, Teucher U, Casson AG (2017). Waiting for scheduled surgery: a complex patient experience. J Health Psychol.

[46] Tod AM, Lacey EA, McNeill F (2002). *'I'm still waiting...': barriers to accessing cardiac rehabilitation services*. J Adv Nurs.

[47] Eve-Lyne Couturier VL (2020). Minh Nguyen, Le revenu viable 2020 dans l'échelle des revenus.

[48] Daniels N, Sabin J (1998). The ethics of accountability in managed care reform. Health Aff (Millwood).

[49] McIntyre D, Chow CK (2020). Waiting time as an Indicator for health services under strain: a narrative review. Inquiry.

[50] Liddy C (2016). Improving access to chronic pain services through eConsultation: a cross-sectional study of the Champlain BASE eConsult service. Pain Med.

[51] Poulin PA (2018). Offering eConsult to Family Physicians With Patients on a Pain Clinic Wait List: An Outreach Exercise. J Healthc Qual..

[52] Haggerty JL (2013). Experienced continuity of care when patients see multiple clinicians: a qualitative metasummary. Ann Fam Med.

[53] Fitzcharles MA, Ste-Marie PA, Pereira JX (2013). Fibromyalgia: evolving concepts over the past 2 decades. Cmaj.

[54] Liddy C (2017). Patient perspectives on wait times and the impact on their life: a waiting room survey in a chronic pain clinic. Scand J Pain.

[55] Rand L (2019). Understanding and using patient experiences as evidence in healthcare priority setting. Cost Effective Resource Allocation.

