# Supplementary material for: The burden of waiting to access pain clinic services: perceptions and experiences of patients with rheumatic conditions
Source: BMC Health Serv Res. 2021 Feb 18;21:160. doi: 10.1186/s12913-021-06114-y (PMC7891805; doi:10.1186/s12913-021-06114-y)
Supplement: Supplementary file 1 — Additional file 1. Table S1. Consolidated criteria for reporting qualitative studies (COREQ): 32-item checklist. [file 12913_2021_6114_MOESM1_ESM.docx]

**Table 1**

Consolidated criteria for reporting qualitative studies (COREQ): 32-item checklist

| **No** | **Item** | **Guide questions/description** | **Response (page)** |
| --- | --- | --- | --- |
| **Domain 1: Research team and reflexivity** | | | |
| Personal Characteristics |  |  |  |
| 1. | Interviewer/facilitator | Which author/s conducted the interview or focus group? | SD and NB (p.7) |
| 2. | Credentials | What were the researcher's credentials? *E.g. PhD, MD* | SD: PhD student NB: undergraduate student (p.7) |
| 3. | Occupation | What was their occupation at the time of the study? | SD: physiotherapist, PhD student  NB: student (p.7) |
| 4. | Gender | Was the researcher male or female? | Both interviewers were males (p.7) |
| 5. | Experience and training | What experience or training did the researcher have? | SD had a specific training in qualitative research; NB was closely supervised by SD p.7 |
| Relationship with participants | | | |
| 6. | Relationship established | Was a relationship established prior to study commencement? | No |
| 7. | Participant knowledge of the interviewer | What did the participants know about the researcher? e*.g. personal goals, reasons for doing the research* | Participant had no prior contact with the interviewers and had no information about them other than their role in the study project |
| 8. | Interviewer characteristics | What characteristics were reported about the interviewer/facilitator? e.g. *Bias, assumptions, reasons and interests in the research topic* | As a physiotherapist, SD had prior experience working with patients referred to pain clinics and having to wait several months/years to receive services. (p. 31-32) |
| **Domain 2: study design** |  |  |  |
| Theoretical framework |  |  |  |
| 9. | Methodological orientation and Theory | What methodological orientation was stated to underpin the study? *e.g. grounded theory, discourse analysis, ethnography, phenomenology, content analysis* | This was a descriptive qualitative design (p.5) |
| Participant selection |  |  |  |
| 10. | Sampling | How were participants selected? *e.g. purposive, convenience, consecutive, snowball* | Convenience, snowball and purposive sampling methods were used (p.6) |
| 11. | Method of approach | How were participants approached? e*.g. face-to-face, telephone, mail, email* | Email or telephone (p.7) |
| 12. | Sample size | How many participants were in the study? | 26 participants (p.9) |
| 13. | Non-participation | How many people refused to participate or dropped out? Reasons? | None |
| Setting |  |  |  |
| 14. | Setting of data collection | Where was the data collected? e*.g. home, clinic, workplace* | In person (home of participant or research centre, according to preference) or over the phone (p.7) |
| 15. | Presence of non-participants | Was anyone else present besides the participants and researchers? | No |
| 16. | Description of sample | What are the important characteristics of the sample? *e.g. demographic data, date* | See p.9-10 |
| Data collection |  |  |  |
| 17. | Interview guide | Were questions, prompts, guides provided by the authors? Was it pilot tested? | The interview guide was pre-tested (p.7) |
| 18. | Repeat interviews | Were repeat interviews carried out? If yes, how many? | No |
| 19. | Audio/visual recording | Did the research use audio or visual recording to collect the data? | Audio recording (p.7) |
| 20. | Field notes | Were field notes made during and/or after the interview or focus group? | Notes were taken after the interviews (e.g., non-verbal signs) |
| 21. | Duration | What was the duration of the interviews or focus group? | 45 minutes on average, ranging from 15 minutes to 2 hours (p.7) |
| 22. | Data saturation | Was data saturation discussed? | Data was collected until data saturation was reached (p.7) |
| 23. | Transcripts returned | Were transcripts returned to participants for comment and/or correction? | No |
| **Domain 3: analysis and findings**z |  |  |  |
| Data analysis |  |  |  |
| 24. | Number of data coders | How many data coders coded the data? | Two coders. SD coded each interviews’ transcript; NB validated the first five and last two interviews (p.8) |
| 25. | Description of the coding tree | Did authors provide a description of the coding tree? | The description of the main themes in the results section reflect the coding tree (p.11-25) |
| 26. | Derivation of themes | Were themes identified in advance or derived from the data? | Content analysis was conducted using both deductive and inductive approaches (p.8) |
| 27. | Software | What software, if applicable, was used to manage the data? | NVivo (p.8) |
| 28. | Participant checking | Did participants provide feedback on the findings? | No |
| Reporting |  |  |  |
| 29. | Quotations presented | Were participant quotations presented to illustrate the themes / findings? Was each quotation identified? e*.g. participant number* | Key quotations were presented and identified with participants’ number (see results section p. 11-25) |
| 30. | Data and findings consistent | Was there consistency between the data presented and the findings? | Yes (see discussion section p.25-31) |
| 31. | Clarity of major themes | Were major themes clearly presented in the findings? | Yes (see discussion section p.25-31) |
| 32. | Clarity of minor themes | Is there a description of diverse cases or discussion of minor themes? | Yes (see discussion section p.25-31) |
