# Supplementary material for: The burden of waiting to access pain clinic services: perceptions and experiences of patients with rheumatic conditions
Source: BMC Health Serv Res. 2021 Feb 18;21:160. doi: 10.1186/s12913-021-06114-y (PMC7891805; doi:10.1186/s12913-021-06114-y)
Supplement: Supplementary file 2 — Additional file 2. Interview guide [file 12913_2021_6114_MOESM2_ESM.docx]

**Interview guide**

(translated from French)

**Section A: General and clinical questions**

1. *First of all, could you please tell me about yourself and the health condition for which you were referred to a pain clinic?*
2. *Could you please tell me about your health condition or rheumatic condition?*

**Section B: Access to pain clinics**

1. *Could you tell me about your care trajectory before being referred to the pain clinic?*
2. *Could you tell me about the waiting period before your first appointment at the pain clinic?*

**Section C: Impacts of the waiting period**

1. *In your opinion, has this waiting period had any consequences in your life or on your condition? If so, what consequences? How did you respond to these consequences?*
2. *During the waiting period, which emotions did you feel?*

*PRN : Phone number of the Centre de crise de Québec, if necessary : 418-688-4240 / 1-866-411-4240

1. *During this waiting period, what strategies did you use to manage your condition?*

**Section D : Outcomes of the services received at the pain clinic**

1. *What were the outcomes of the services received at the pain clinic?*
2. *What were your expectations regarding the services delivered at the pain clinic?*
3. *To what degree the services delivered at the pain clinic met your expectations?*

**Section E: Solutions to improve access**

Now, I would like you to think about possible solutions to improve access to pain clinics.

1. *In your opinion, could something have been done:*
   1. ***at the moment of the referral*** *to a pain clinic in order to improve your experience?*
   2. ***during the waiting period*** *before receiving the services from the pain clinic in order to improve your experience?*
   3. *to help you relieve your pain during the waiting period?*

**Section F: Conclusion**

1. *Would you like to add anything else regarding the issue of access to pain clinics?*
2. *Any other thoughts you would like to share with me?*
